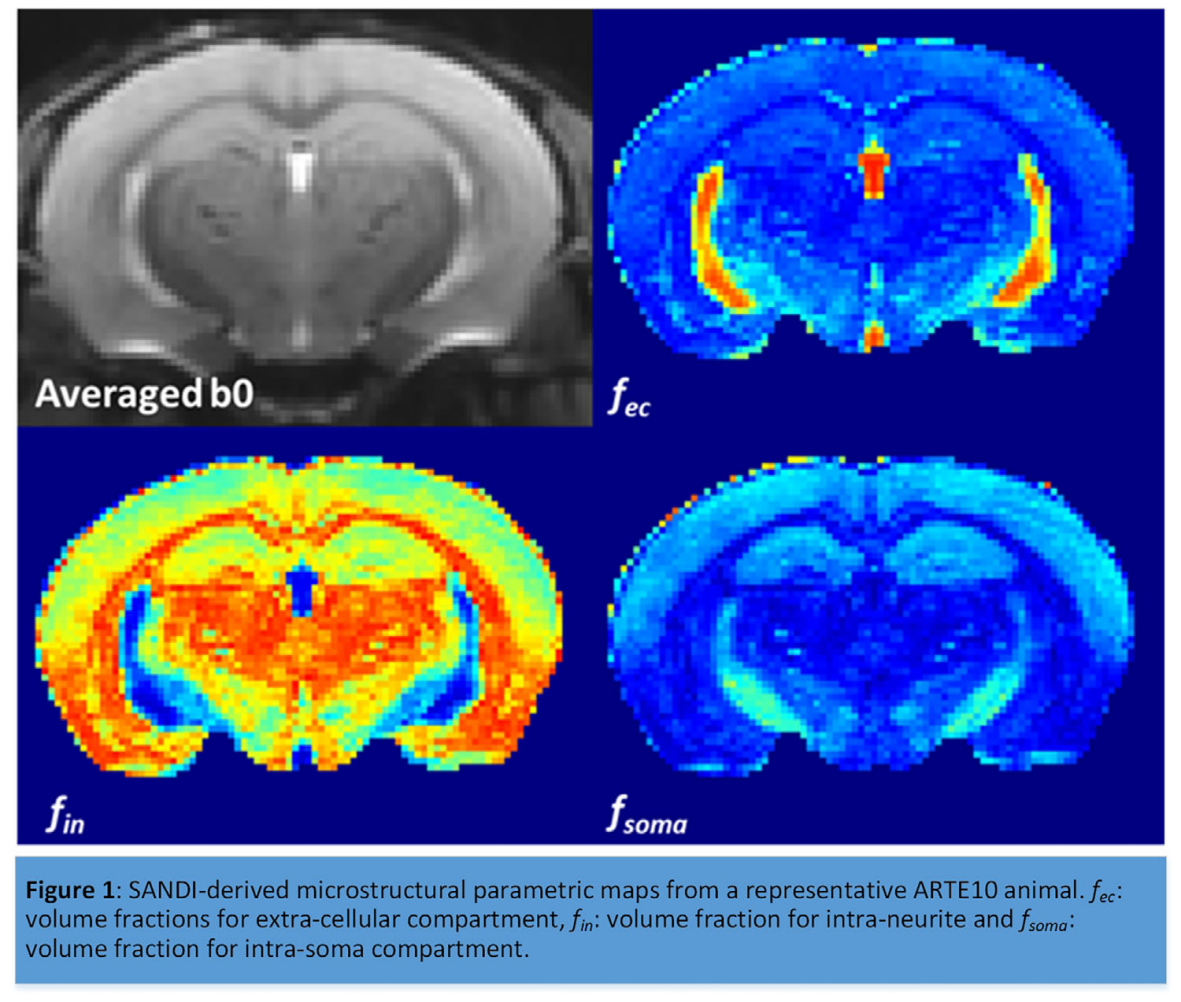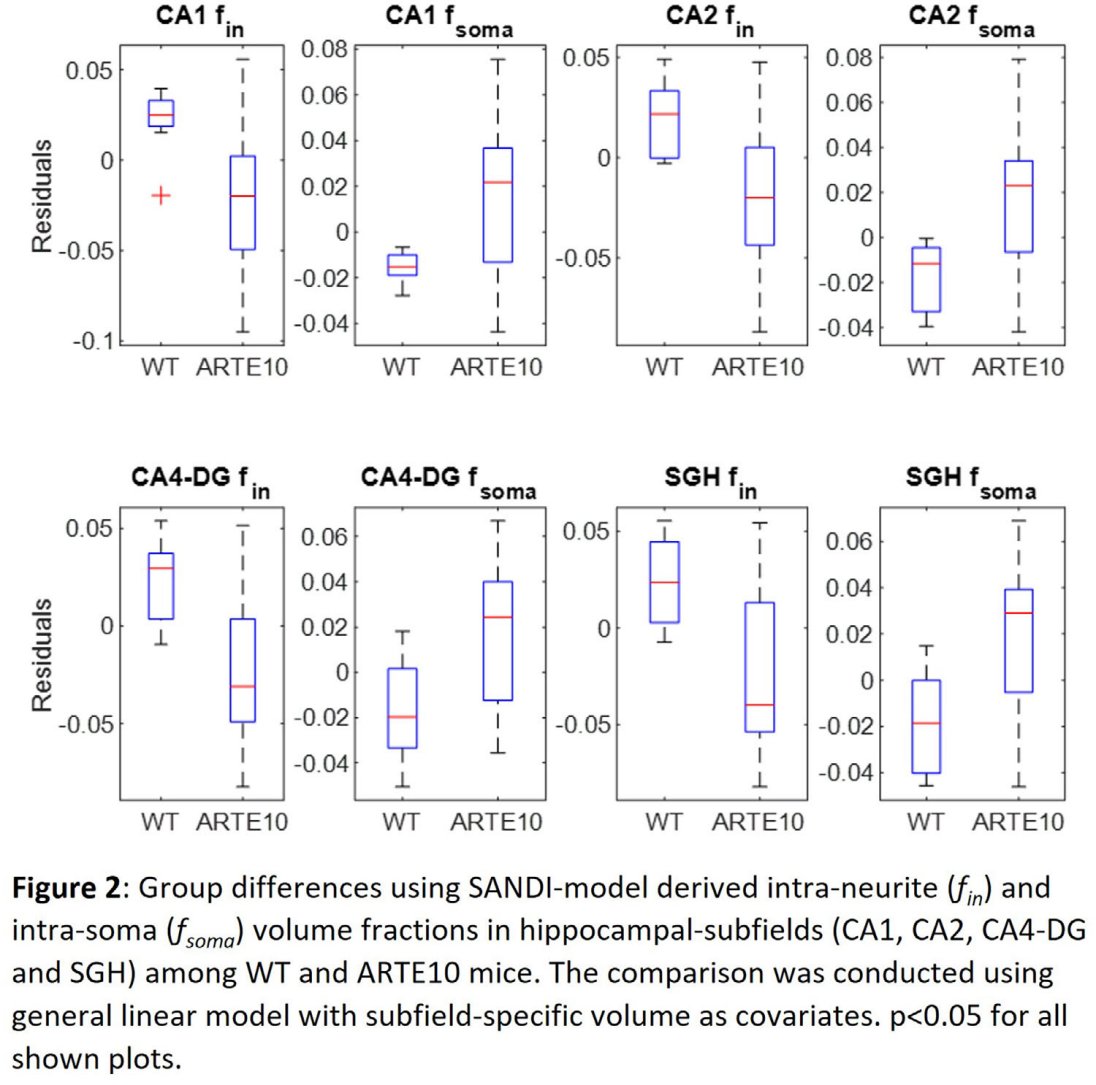# Mapping alterations in the hippocampal subfields in the ARTE10 mouse model of Alzheimer's disease using dMRI derived soma and neurite density maps

**DOI:** 10.1002/alz.093865

**Published:** 2025-01-09

**Authors:** Syed Salman Shahid, Erin E Jarvis, Elizabeth R Butch, Scott E Snyder, Yu‐Chien Wu

**Affiliations:** ^1^ Indiana University School of Medicine, Indianapolis, IN USA

## Abstract

**Background:**

The hippocampus, a region vital for memory and cognition, is prone to abnormal deposition of beta‐amyloid (Aß) during the early stages of the Alzheimer’s disease. Aß‐associated pathophysiological mechanisms instigate dendritic deficit, neuronal loss, and neuroinflammation, leading to abnormal functional and behavioral changes. These factors directly impact tissue microstructures. Histological studies suggest subfield specific Aß accumulation. The aim of the current study is to map the changes in soma‐ and neurite‐density in the hippocampal‐subfields due to Aß load in the ARTE10 mouse‐model of AD using a compartment‐specific diffusion model.

**Method:**

Ten‐month‐old male ARTE10 (N=8) and age‐matched littermate controls underwent MRI on a 9.4T scanner (Bruker BioSpin, GmbH, Germany) equipped with an 1H cryogenic surface coil. DWIs were acquired using a 2D multi‐shot spin‐echo echo planar imaging sequence using the following parameters: TE/TR=26/4000 ms; voxel size=125x125x400 µm3, 20 slices, 4 segments, b values= (500, 1000, 2500, 4000, 5500, 7000) s/mm2 and 5 b0 per b‐shell. The averaged b0 image was non‐linearly registered to Badhwar hippocampus atlas. The hippocampal‐subfields (CA1, CA2, CA3, CA4‐DG, subiculum, and stratum‐granulosum of hippocampus (SGH)) in atlas space were inverse transformed to diffusion space. Multi‐compartment microstructural estimation was performed using soma and neurite density imaging (SANDI) model. This model provided volume fractions for extra‐cellular (fec), intra‐neurite (fin), and intra‐soma (fsoma) compartments (Figure 1). The regional mean values of microstructural parameters of individual subfields were extracted and used in a general linear model for group comparisons.

**Result:**

The group‐level differences (WT vs. ARTE10) in fin were observed in CA1 (p=0.02; d=‐1.25), CA2 (p=0.03; d=‐1.20), CA4‐DG (0.01; d=‐1.34), and SGH (p=0.03; d=‐1.22) Figure 2. Group differences in fsoma were significant in CA1 (p=0.04; d=1.08), CA2 (p=0.02; d=1.22), CA4DG (p=0.04; d=1.10), and SGH (p=0.03; d=0.99). In general, the ARTE10 AD mice had reduced neurite density and elevated soma density in the hippocampal subfields.

**Conclusion:**

The results demonstrated that the SANDI model is sensitive to the microstructural alterations in the hippocampal subfields of the animal model with profound AD pathologies.